# Erosive oral lichen planus

**DOI:** 10.11604/pamj.2021.40.73.26013

**Published:** 2021-10-04

**Authors:** Pragadeesh Palaniappan, Krishna Prasanth Baalann

**Affiliations:** 1 Department of Community Medicine, Sree Balaji Medical College and Hospital, Bharath Institute of Higher Education and Research (BIHER), Chennai, India

**Keywords:** Erosive lichen planus, premalignant, oral lichen planus

## Image in medicine

Plethora of clinical forms exists in oral lichen planus (OLP), a common autoimmune disease involving mucous membrane. The commonest forms described in the literature are reticular, atropic, papular, bullous, plaque and erosive. The erosive type being the second most common, is a premalignant condition owing to its aggressiveness. A 54-year-old male presented to private clinic with one year history of intermittent burning sensation on inner aspect of cheeks. He was on antihypertensives since two years and using tobacco for the past ten years. On oral examination, the lesion was extending from commissure of lip to retromolar region bilaterally, with elevated white lacy streaks/striae (Wickam´s striae) throughout the lesion, typical of lichen planus (LP). On palpation, tenderness (+) and lesion was unscrapable. The case was provisionally diagnosed as erosive LP. Incisional biopsy was taken and histopathological examination confirmed the provisional diagnosis. Patient was treated with antioxidants, topical and systemic steroids. After one week, patient had a note-worthy reduction in burning sensation. Patient was encouraged to quit tobacco, educated about oral cleanliness and was encouraged to seek regular follow-up to monitor the disease. The odds of oral lichen planus developing into squamous cell carcinoma go up to 10, among which erosive type has a noteworthy frequency. Hence, it is prudent for all clinicians to do oral examination for each and every patient for it provides a clue to diagnose a wide gamut of diseases. Early identification of oral lesions will help in effective treatment as well as to avert grievous complications like malignant transformation.

**Figure 1:**
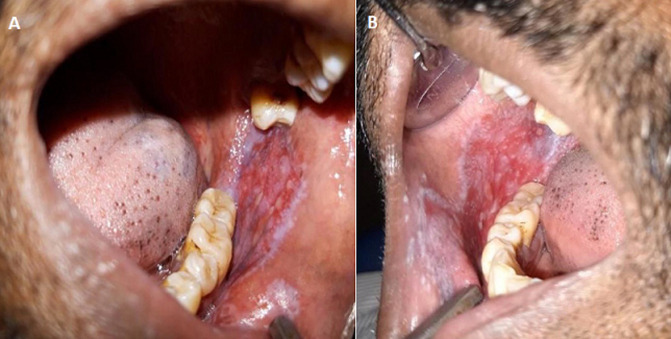
(A,B) lesion extending from commissure of lip to retromolar region bilaterally, with elevated white lacy streaks/striae (Wickam´s striae) throughout the lesion, typical of lichen planus

